# Supplementary material for: Towards Machine Learning in Heterogeneous Catalysis—A Case Study of 2,4-Dinitrotoluene Hydrogenation
Source: Int J Mol Sci. 2023 Jul 14;24(14):11461. doi: 10.3390/ijms241411461 (PMC10380742; doi:10.3390/ijms241411461)
Supplement: Supplementary file 1 [file ijms-24-11461-s001.zip › ijms-2427562-supplementary.pdf]

Supplementary Information

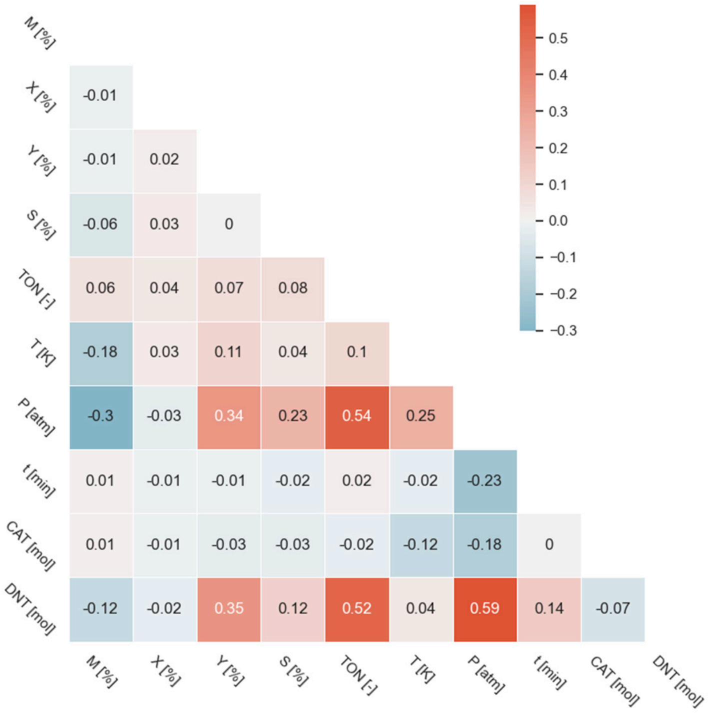

S1. Filtered dataset of statistical analysis according to MIRA21

Dataset of statistical analysis according to MIRA21[35–49]

MAIN_A_COMP	No.A_COMP	A_METALCONT	CARRIER	Solvent	MAXCONV	PRYIELD	PRSEL	TON	TEMP	PRESS	TIME	nCAT	nDNT
		w/w%			%	%	%	-	K	atm	min	mol	mol
Platinum	1	5.0	TMO	methanol	100	62	62	181	333	20	60	2.56E-05	0.008
Palladium	1	5.0	TMO	methanol	100	83	83	132	333	20	60	4.70E-05	0.008
Palladium	2	4.5	TMO	methanol	100	87	87	145	333	20	30	4.49E-05	0.008
Palladium	1	4.5	TMO	methanol	100	100	100	176	333	20	80	4.25E-05	0.008
Platinum	1	3.0	TMO	methanol	100	99	99	479	323	20	40	1.55E-05	0.008
Platinum	1	0.2	TMO	ethanol	100	97	97	6661	353	10	29	1.60E-06	0.011
Platinum	1	0.2	TMO	ethanol	100	99	99	6784	353	10	21	1.60E-06	0.011
Platinum	1	0.2	TMO	ethanol	100	98	98	6740	353	10	24	1.60E-06	0.011
Platinum	1	0.4	TMO	ethanol	100	98	98	2389	353	10	13	4.52E-06	0.011
Platinum	1	0.5	TMO	ethanol	100	98	98	2082	353	10	12	5.19E-06	0.011
Platinum	1	0.5	TMO	ethanol	100	98	98	2004	353	10	12	5.35E-06	0.011
Palladium	1	0.5	Carbon	ethanol	100	83	83	445	323	1	19	4.68E-06	0.003

Palladium	1	1.0	Carbon	ethanol	100	82	82	196	323	1	20	1.05E-05	0.003
Palladium	1	1.0	Carbon	ethanol	100	93	93	210	323	1	12	1.11E-05	0.003
Palladium	1	1.0	Carbon	ethanol	100	87	87	235	323	1	7	9.29E-06	0.003
Palladium	1	1.0	Carbon	ethanol	100	84	84	232	323	1	98	9.04E-06	0.003
Palladium	1	3.0	Carbon	ethanol	100	82	82	84	323	1	71	2.44E-05	0.003
Palladium	1	5.0	Carbon	ethanol	100	93	93	100	323	1	52	2.32E-05	0.003
Palladium	1	5.0	Carbon	ethanol	100	97	97	119	323	1	87	2.04E-05	0.003
Palladium	1	3.8	TMO	methanol	100	100	100	111	333	20	40	3.82E-05	0.010
Palladium	1	4.0	TMO	methanol	100	84	84	91	333	20	40	3.98E-05	0.010
Palladium	1	4.3	TMO	methanol	100	54	54	27	333	20	120	4.08E-05	0.010
Iridium	3	5.0	Carbon	methanol	100	99	99	6950	393	50	50	3.93E-05	0.275
Iridium	3	5.0	Carbon	methanol	100	100	100	6957	393	50	45	3.93E-05	0.275
Palladium	1	5.0	Carbon	methanol	100	98	98	4591	393	50	30	5.87E-05	0.275
Palladium	2	2.0	Carbon	methanol	100	99	99	2322	393	50	60	1.17E-04	0.275
Palladium	2	2.0	Carbon	methanol	100	98	98	2308	393	50	40	1.17E-04	0.275
Nickel	1	20.0	Zeolite	methanol	100	85	85	21	348	26	120	1.70E-03	0.041
Nickel	1	10.0	Zeolite	methanol	100	78	78	19	348	26	120	1.70E-03	0.041
Palladium	1	5.1	TMO	methanol	100	99	100	158	333	20	120	4.75E-05	0.008
Palladium	1	5.3	TMO	methanol	99	66	66	100	333	20	240	4.93E-05	0.008
Palladium	1	4.0	TMO	methanol	100	100	100	201	333	20	120	3.72E-05	0.008
Palladium	1	4.3	Carbon	methanol	100				373	14	14	4.28E-05	0.100
Palladium	1	3.8	Carbon	methanol	100				373	14	10	4.28E-05	0.100
Palladium	1	4.3	Carbon	methanol	100				373	14	9	4.28E-05	0.100
Palladium	1	4.0	Carbon	methanol	100				373	14	16	4.28E-05	0.100
Palladium	1	4.4	Carbon	methanol	100				373	14	12	4.28E-05	0.100
Palladium	1	4.4	Carbon	methanol	100				373	14	9	4.28E-05	0.100
Palladium	1	4.7	Carbon	methanol	100				373	14	6	4.28E-05	0.100
Palladium	1	4.5	Carbon	methanol	100				373	14	7	4.28E-05	0.100
Nickel	1	39.7	Zeolite	ethanol	100	99	99	8	363	22	75	3.38E-03	0.027
Palladium	2		Polymer	ethanol	78				338	1	120	4.00E-06	0.002
Palladium	2		Polymer	ethanol	99	99	100	1000	338	1	37	4.00E-06	0.004
Nickel	3	68.6	None		95		100		383	20	120	5.84E-03	0.027
Nickel	3	67.9	None		98		100		383	20	110	5.78E-03	0.027
Nickel	3	67.4	None		100		100		383	20	100	5.74E-03	0.027
Nickel	3	63.4	None		100		100		383	20	60	5.40E-03	0.027
Nickel	3	58.9	None		100		100		383	20	80	3.39E-02	0.027

TMO - transition metal oxide, **PTMO -** post transition metal oxide

S2. Results of Spearman correlation analysis with full data set Differences of the Unfiltered and filtered data set results

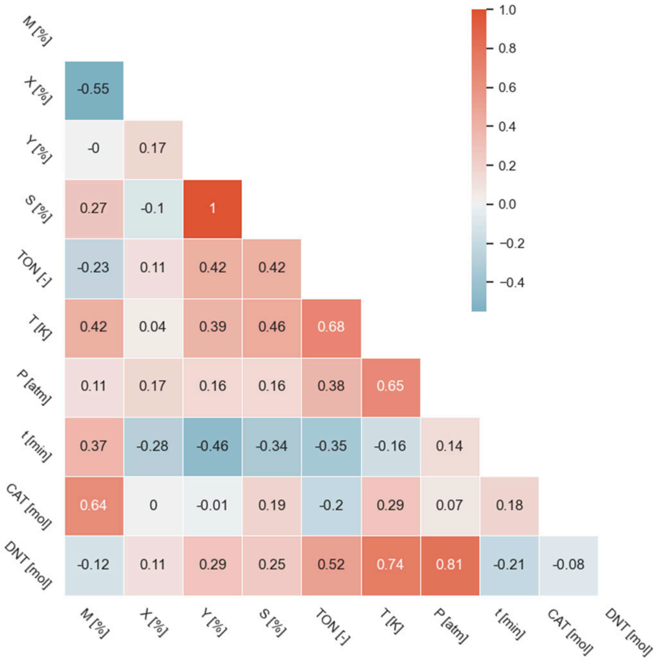

S3. Results of Spearman correlation analysis – differences of the unfiltered and filtered data set results
